# Realized niche shift of an invasive widow spider: drivers and impacts of human activities

**DOI:** 10.1186/s12983-022-00470-z

**Published:** 2022-10-28

**Authors:** Zhenhua Luo, Monica A. Mowery, Xinlan Cheng, Qing Yang, Junhua Hu, Maydianne C. B. Andrade

**Affiliations:** 1 Institute of Evolution and Ecology, School of Life Sciences, Central China Normal University, Wuhan, 430079 Hubei China; 2 Mitrani Department of Desert Ecology, The Jacob Blaustein Institutes for Desert Research, Ben-Gurion University of the Negev, 8499000 Midreshet Ben-Gurion, Israel; 3 Department of Biological Sciences, University of Toronto Scarborough, Toronto, ON M1C 1A4 Canada; 4 Xiamen No 3. Middle School of Fujian, Xiamen, 361006 Fujian China; 5 Chengdu Institute of Biology, Chinese Academy of Sciences, Chengdu, 610041 Sichuan China

**Keywords:** Australian redback spider, Ecological niche model, Invasive species, *Latrodectus hasselti*, Niche change

## Abstract

**Background:**

Predicting invasiveness requires an understanding of the propensity of a given species to thrive in areas with novel ecological challenges. Evaluation of realized niche shift of an invasive species in its invasive range, detecting the main drivers of the realized niche shift, and predicting the potential distribution of the species can provide important information for the management of populations of invasive species and the conservation of biodiversity. The Australian redback spider, *Latrodectus hasselti*, is a widow spider that is native to Australia and established in Japan, New Zealand, and Southeast Asia. We used ecological niche models and ordinal comparisons in an integrative method to compare the realized niches of native and invasive populations of this spider species. We also assessed the impact of several climatic predictor variables and human activity on this niche shift. We hypothesized that human impact is important for successful establishment of this anthropophilic species, and that climatic predictor variables may determine suitable habitat and thus predict invasive ranges.

**Results:**

Our models showed that *L. hasselti* distributions are positively influenced by human impact in both of the native and invasive ranges. Maximum temperature was the most important climatic variable in predictions of the distribution of native populations, while precipitation seasonality was the most important in predictions of invasive populations. The realized niche of *L. hasselti* in its invasive range differed from that in its native range, indicating possible realized niche shift.

**Conclusions:**

We infer that a preference for human-disturbed environments may underlie invasion and establishment in this spider species, as anthropogenic habitat modifications could provide shelters from unsuitable climatic conditions and extreme climatic stresses to the spiders. Because Australia and the countries in which the species is invasive have differing climates, differences in the availability of certain climatic conditions could have played a role in the realized niche shift of *L. hasselti*.

**Supplementary Information:**

The online version contains supplementary material available at 10.1186/s12983-022-00470-z.

## Background

Since Hutchinson’s concept of the environmental niche was proposed [1], one of the classic questions in ecology and evolutionary biology has been how environmental conditions determine species' distributions [2–5]. Two kinds of environmental niches have been evaluated in previous studies. The fundamental niche depicts a species’ ecophysiological requirements, which is the envelope of environmental conditions (a multi-dimensional environmental space) in which individuals can survive. The realized niche (ecological niche) is the environmental niche of a species as quantified from field observations, which is the environmental envelope modulated from the fundamental niche by biotic exclusions, population dynamics, and dispersal limitations [2]. Although it is possible to measure the fundamental niche based on physiological information and experiments for some species, for most species, only the realized niche can realistically be estimated through empirical studies [2, 6–10]. It has become increasingly important to describe and understand the extent to which species’ realized niche can change rapidly (i.e., niche shift) or remain stable (i.e., niche conservatism) in the context of ongoing global change [4]. Based on the information, it is possible to predict the distribution of a species at present and potential distribution changes under future climate change [2–7]. Thus, comparing the differences and dynamics of species’ realized niches between different geographic areas or time periods, across a range of different taxa, is urgently needed to support a broader understanding of these phenomena, particularly for invasive species [2, 4, 7, 11].

Biological invasions provide good opportunities to explore the mechanisms important during the colonization of new environments, and to investigate whether invasive species retain their realized niches when introduced to new areas with environmental conditions that differ from their native ranges [3, 12–14]. Previous reports on terrestrial plants, invertebrates, fish, amphibians, reptiles, and birds concluded that realized niche shifts are rare overall between native and invaded ranges [8, 13, 15–20]. However, the assumption of realized niche conservatism has been challenged by increasing evidence of shifts from several taxonomic groups in recent years (e.g., plants, insects, aquatic invertebrates, aquatic vertebrates, mammals) [15, 21–28]. The generality of conclusions about realized niche shift are unclear since studies of invasive species are heavily biased towards relatively few taxa (e.g., over 60% on insects and plants [11]). Understanding whether invasive species will typically be restricted in their new ranges by realized niche conservatism, or whether shifts may allow broader distributions is important to conservation efforts. That is because, the invasive range predicted based on observation data from native range will differ under different pivotal assumptions (niche conservatism vs. niche shift) [21]. As one of the major drivers of global change, introduction and spread of invasive species can trigger biodiversity loss and ecosystem disruption in various ways [25, 29]. This may include agricultural and forestry losses through reduced yields and pathogen transmissions [30], and negative effects on human well-being due to some invasive species being vectors of human diseases (such as the mosquito *Aedes aegypti* [31]). Therefore, exploring how realized niche shifts may be part of the invasion process, and how this may impact predictions of distribution and spread, can be key to informing spatial prioritization, early detection and control, and management policies against biological invasions [25, 32, 33].

There are a number of considerations necessary when seeking to determine whether realized niche shifts have occurred. First, the most commonly used metrics measuring differences between realized niches are changes to the realized niche centroid, measured using the Euclidean distance between the mean positions of the native and invasive realized niche spaces [21], and the degree of realized niche overlap, such as Schoener’s *D* index [34–36]. However, an absence of a realized niche centroid shift does not necessarily indicate that there has been no realized niche shift [2]. A change of the realized niche envelope might occur without a shift in the centroid as a result of symmetric expansion, contraction, or displacement within environmental space. For example, a move to both warmer and colder or wetter and drier conditions may occur in such a way that the average realized niche positions remain stable [2, 17, 18, 21, 37, 38]. Thus, it is more appropriate to use more comprehensive measures of realized niche shift. Guisan et al. [2] developed a framework that decomposes realized niche shift into three situations. That is, unfilling (proportion of the native realized niche that does not overlap with the invasive realized niche), stability (proportion of the invasive realized niche overlapping with the native realized niche), and expansion (proportion of the invasive realized niche that does not overlap with the native realized niche). In addition, demonstrating meaningful realized niche shifts may depend on choosing biologically relevant environmental variables for analysis [39, 40], and mapping the availability of environmental conditions in geographic space could also be crucial for exploring realized niche differences between native and invasive ranges of an alien species [2]. This is because some environmental conditions that are common in the native range might be rare in the invasive range, and vice versa, because of the niche-biotope duality [41]. This may produce results that indicate realized niche shift, but in reality these differences would arise from differences in the occurrence of conditions in different parts of the species range, even if these do not impact the success of the species under study [5, 38]. Therefore, for a robust understanding of realized niche shift of an invasive species, realized niche centroid dynamics and the three niche shift elements proposed by Guisan et al. [2] should be measured, while also considering the relative availability of multiple environmental conditions with an understanding of how these are biologically relevant to the species under study. We used this approach to study the realized niche of the invasive Australian redback spider (*Latrodectus hasselti*).

Generally, two approaches have been used to analyze realized niche shifts of invasive species: comparisons of environmental attributes of native and invasive ranges based on direct observations of environmental conditions at sites where the organism occurs in these ranges and detecting the overlaps of reciprocal predictions of native and invasive geographical distributions based on the outcomes of ecological niche models (ENMs; also named species distribution models, SDMs) [2, 4, 7, 34, 36, 42]. The first method uses univariate or multivariate tests or principle component analysis (PCA), an ordinal way to quantify realized niche difference, which has higher accuracy overall than the second method. However, this approach provides a less mathematical representation of the realized niche than the ENM method, and does not allow for weighting predictor variables based on their importance in the species’ ecology [2, 26, 42]. The ENM method predicts the native and invaded distributions with models fitted based on occurrence records from field observations, and then compares the situations of the two predictions [36, 42, 43]. It is a visualized way to detect realized niche shift and is particularly useful in assessing ENM transferability between native and invasive ranges [44]. Here, we take an integrated approach that utilizes the strengths and minimizes the weakness of each method by conducting ordinal analyses based on the results from ENMs to quantify realized niche shifts [2, 34].

In addition to a focus on environmental factors, we also examine effects of human activities in our models. Human activities have obvious impacts on many ecological processes, and on the distributions of animals and plants at different spatial and temporal scales, and this is particularly true for invasive species [25, 45–47], many of which are adapted to human-disturbed environments [47–50]. It has been suggested that alien species usually establish in disturbed areas at the early stages of the invasion process before range expansion [51]. Thus, anthropogenic impacts can affect the realized niche of invasive species and should be included as a predictor of the distributions of invasive species [25, 45, 47]. There is some evidence that adding human footprint to climatic variables can improve predictions (e.g., in terrestrial plants [52]). Nevertheless, the role of human disturbance in mediating realized niche shifts has received much less attention than other factors [18, 53].

The widow spiders (genus *Latrodectus*) include over 30 species found around the world [54] with medically important, neurotoxic venom [55], which makes invasive populations a particular concern [56]. Spiders in this genus are generalist predators that can survive for months without food, have a high reproductive output [57, 58], and may be easily introduced to new areas by human transport [59, 60]. Nevertheless, to date only two species have been reported to be invasive (*L. hasselti* and *L. geometricus* [54]). Australian redback spiders (*L. hasselti,* Thorell 1870, Araneae: Theridiidae) is native to Australia, where it is common in urban habitats, and it has established populations in New Zealand (first recorded in 1981–1982 [61, 62]), Japan (first recorded in 1995 [63]), India [64], and Southeast Asia (the Philippines [65]), likely through international cargo shipments of steel, produce, or wood [66]. Here we examine the realized niches of native and invasive populations of *L. hasselti* at a continental scale using ENM and ordinal comparisons combined with considerations of environmental availability, and including analyses of realized niche centroid change, unfilling, stability, and expansion. In addition, we hypothesized that human impact supports the successful establishment of this anthropophilic species [66]. Thus, we also compare the influence of human disturbance on native and invasive populations. We predicted the potential distributions of *L. hasselti* in its native and invasive ranges and detected the main drivers of its realized niche shift. We hope this study can provide data for population management of this species, and provide reference to prioritization, early detection, control, and management policies against the invasion.

## Materials and methods

### Natural history

Past work suggests that, for *Latrodectus hasselti*, variation in temperature and precipitation are important determinants of environmental suitability, range extent, survival, growth, and offspring development [61, 66–69]. Although *L. hasselti* spiders can tolerate a wide range of temperatures, their growth may be slowed and egg sacs may cease development at low temperatures [70]. Matsuse et al. [68] reported that the greatest survival rate of the spiders was at 20 °C; if under 5–10 °C, they could survive for a month but did not grow or moult. Juvenile spiders can survive short exposure (20 min) to freezing temperatures, however, the developmental zero of the egg sacs (i.e., the temperature at which egg sac development ceases) is 15–18 °C and the spiderlings do not emerge at 20 °C [68, 69]. In addition to these effects of temperature, *L. hasselti* are adapted to xeric conditions, so may not be able to survive in areas with high precipitation or humidity [61]. *Latrodectus hasselti* forage less actively on rainy days, and flooding may destroy webs, both of which could negatively impact survival (our field observations), although protected microhabitats may provide protection against the rain for cobweb spiders [71], and such protection may be abundant in human-disturbed habitats [66].

### Data collection and realized niche modeling

**Fig. 1:**
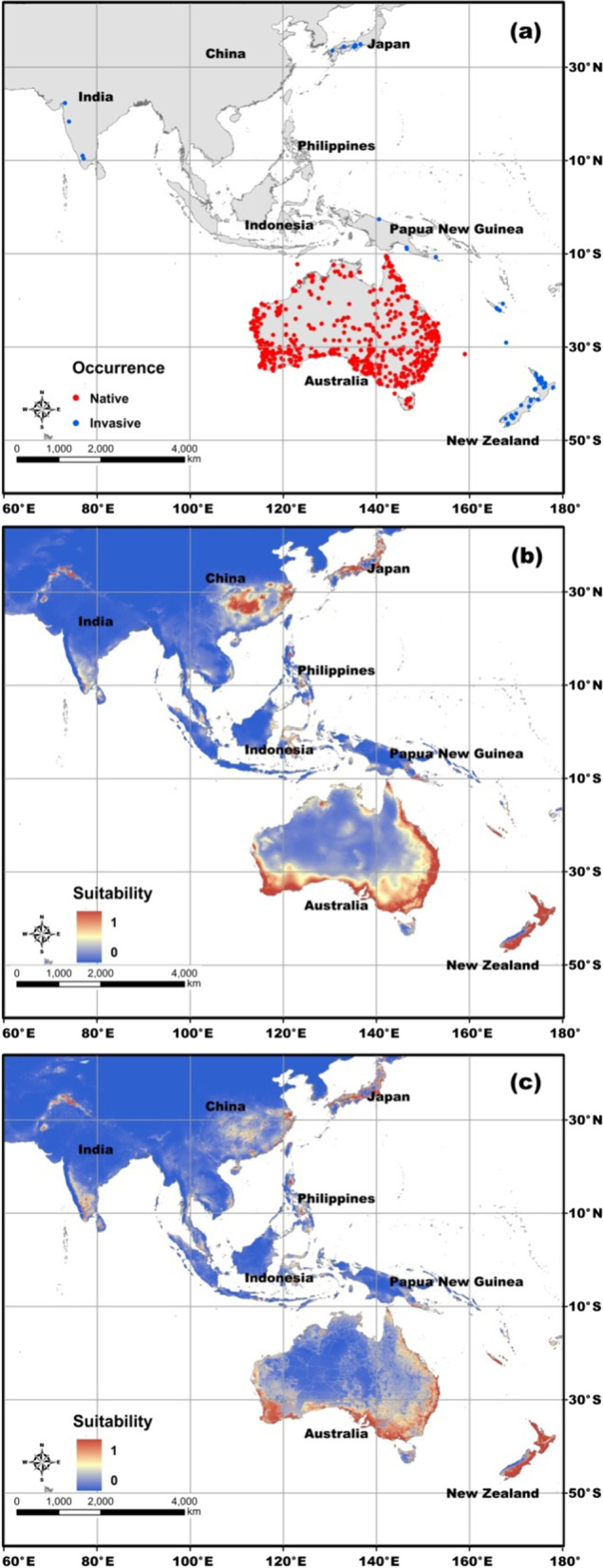
Study area, occurrence records, and habitat suitability of *Latrodectus hasselti*. **a** Map of the study area, with native occurrence records (AUS) shown in red and invasive occurrence records (INV) shown in blue. **b** Habitat suitability patterns based on climatic models. **c** Habitat suitability patterns based on full models. Habitat suitability patterns in Australia and countries other than Australia were processed from AUS and INV models, respectively

The study area was across eastern and southern Asia and Oceania (45° N–60° S, 60°E–180°; Fig. 1).

We searched for occurrence records of *L. hasselti* in the Global Biodiversity Information Facility (GBIF, http://www.gbif.org/, [72]) using its scientific name (‘*Latrodectus hasselti*’) and English name (‘Australian redback spider’), and also the synonyms of ‘redback spider’, and ‘redback widow spider’, ‘*Latrodectus scelio*’, ‘*Latrodectus indicus*’, ‘*Latrodectus ancorifer*’, and ‘*Latrodectus mactans*’. Searches for records were also carried out in Google Scholar for localities of the species in published literature. A total of 2580 raw occurrence records (GPS localities) of *L. hasselti* were collected from the GBIF and literature [66, 68, 73] for the study area. We compiled these location points into two subsets: native occurrences (Australia; 1967 localities) and invasive occurrences (other countries; 613 localities). We used the CoordinateCleaner package to remove records located at sites where museums, institutes, and capital cities are found [74]. We deleted the records with latitude/longitude coordinates with less than two decimal places because of their coarse resolution (models were at a 1 km × 1 km resolution, see below). To avoid geo-referencing errors and over-fitting the models, we checked locations of the occurrence records in ArcGIS 10.0 (ESRI, Redlands, USA) and removed duplicate occurrences at a spatial resolution of 1 km × 1 km so that each grid cell had only a single record [75–79]. Finally, 859 and 82 occurrence records for Australia and the other countries, respectively, were used for modeling (Fig. 1a; Table S1 in Additional File 1).

Based on previous research [66] and our field observations, seven climatic parameters, which are the most ecologically relevant to survival, habitat use, and fitness of *L. hasselti*, were selected to construct ecological niche models: annual mean temperature (AMT), mean diurnal temperature range (MDR), temperature seasonality (TS), maximum annual temperature (MaxT), minimum annual temperature (MinT), annual precipitation (AP), and precipitation seasonality (PS). Raster data of these variables (1 km × 1 km) were extracted from the WorldClim V2 database [80, 81] and transformed into equal-area grids in ArcGIS 10.0 with an UTM WGS 1984 projection. As an estimate of human impact, we used the human footprint (HFP, 1 km × 1 km) data from Last of the Wild Data Version 2 [82] and Venter et al. [83] and transformed it into an equal-area raster with an UTM WGS 1984 projection. HFP is an index that integrated from nine human influence datasets, covering human population pressure (population density), human land use and infrastructure (built-up areas, nighttime lights, land use, land cover), and human access (coastlines, roads, railroads, navigable rivers). Then, all the data were split into two subsets: native (Australia) and invasive (other countries). Modelling was performed at the highest resolution (1 km × 1 km) at which the continental scale predictor variables were available. We extracted the values of each environmental variable by grid cell and checked for co-linearity between variables using Pearson’s correlation analysis. As no correlation coefficient of > 0.7 was detected, all the environmental variables were included in the models.

Using a maximum entropy approach in MaxEnt v3.3.3 k [84–86], we predicted the potential distribution of *L. hasselti* in Australia (native distribution; AUS model) using the occurrence records and climatic predictor variables from the native range, and predicted the potential distribution of *L. hasselti* in other countries (invasive distribution; INV model) using occurrence records and climatic predictor variables from the invasive range. MaxEnt has been shown to have good performance which consistently outperforms many other methods, especially when sample sizes are small, and input data are noisy, and is robust to various levels of correlation among parameters [84, 87]. To explore the impacts of human activities on the spider’s distribution, a climatic model (using the seven climatic variables) and a full model (using the seven climatic variables and HFP) were processed simultaneously for both the native and invasive ranges. As sampling bias in background points could decrease modeling effectiveness [84], we used a Kernel Density Estimator (KDE) surface to create 10,000 background points throughout the study area using the Software for Automated Habitat Modeling (SAHM) [88–90]. This technique was used because randomly sampled background points may not represent the entire range of environmental conditions in the study area and might underrepresent/overrepresent some environmental conditions, KDE can generate background points with a smoothed density across the environmental space for each environmental factor [34]. These background points were split into two subsets: native (background points in Australia) and invasive (background points in other countries), and used for the AUS model and INV model, respectively. To avoid reduction of model accuracy caused by an inappropriate model complexity or data organization, we selected model settings and conducted model selection by following Muscarella et al. [91] using the ENMeval 0.1.0 package in R 4.0.2 [92, 93]. That is, models were built using a combination of five feature class values (FC: linear, quadratic, product, threshold, hinge), eight regularization multiplier values (RM: 0.5, 1, 1.5, 2, 2.5, 3, 3.5, 4), and four locality partition methods (randomkfold, block, checkerboard1, checkerboard2). The mean values of the area under the receiver operating characteristics curve using the testing data (mean AUC_test_) and the mean values of the difference between the training and testing AUCs (mean AUC_diff_) were computed as these metrics give an indication of the goodness-of-fit and degree of overfitting of the model. We used the Akaike Information Criteria corrected for a small sample size (AICc) and AICc weight (AICw) to compare different parameter combinations and select models [93, 94]. The models incorporating all environmental parameters (i.e., seven climatic variables for the climatic model, seven climatic variables and HFP for the full model) and with FC = linear, RM = 1, and the randomkfold (k = 10) partition method performed best for both of the AUS and INV models (with the lowest AICc and the highest AICw, see Results; Table. S2–17 in Additional File 2). Other default settings of the software were adopted: maximum iterations (500), and convergence threshold (10^−5^) [84]. We selected logistic output format (habitat suitability value ranges from 0 to 1) and conducted jackknife procedures to evaluate relative contribution of each variable to the model [84]. We ran ten cross-validation replications of the model and weighted them by their AUCs to obtain an ensemble distribution prediction [95, 96]. To convert continuous outputs into binary maps (suitable or unsuitable), we extracted the suitability estimates from the models at the locations of the occurrence records, calculated the mean suitability, and used these values as thresholds (AUS climatic model: 0.702, AUS full model: 0.663; INV climatic model: 0.681, INV full model: 0.630) [97, 98]. That is, areas with a predicted suitability above and below the threshold were respectively considered as suitable and unsuitable for *L. hasselti*. The proportion of omission errors (occurrence records that were found in unsuitable sites) on these thresholds were 8.5% for the AUS climatic model, 9.1% for the AUS full model, 3.7% for the INV climatic model, and 4.9% for the INV full model. As the omission error rate was low for all models, we regarded this as a suitable threshold.

In addition, we built and evaluated two models based on all the occurrence records of *L. hasselti* and environmental predictor variables in the study area using the same model settings as the AUS and INV models (FC = linear, RM = 1, partition method = randomkfold (k = 10)). That is, a climatic model based on all the occurrence records and the seven climatic predictor variables in Australia and the other countries and a full model based on all the occurrence records and the seven climatic predictor variables and HFP in Australia and the other countries were developed, respectively.

### Quantifying realized niche differences

We extracted the climate data for each climate variable at the sites predicted to be suitable by the climatic models, and compared the means between AUS and INV using one-way ANOVAs. To explore centroid shift, a principle component analysis (PCA) was processed in the “ade4” library in the R 4.0.2 software [99]. We compared the climatic spaces of the native and invaded distributional ranges of the spiders, and the magnitude and statistical significance of the realized niche shift evident in the PCA graph were computed by a between-class analysis with a between-class inertia percentage [21]. Nine hundred and ninety-nine Monte-Carlo randomizations were conducted to test this ratio [100]. We also calculated the Schoener’s *D* and Hellinger’s *I* in ENMTools to show the climatic realized niche similarity [43]. Both indexes vary between 0, when populations’ predicted niches do not overlap at all, and 1, when all predicted environments are equally suitable for both populations. Then, we extracted the data of each climate variable for Australia and the other countries across the study area by grid cell, and calculated three niche shift elements of each variable (AUS vs. INV): unfilling (proportion of AUS realized niche that does not overlap with INV realized niche; ranges from 0, when all predicted suitable environments for the native population are occupied by the invasive population, to 1, when no predicted suitable environment for the native population is occupied by the invasive population), stability (proportion of INV realized niche overlapping with AUS realized niche; ranges from 0, when no predicted suitable environment for the invasive population is suitable for the native population, to 1, when all predicted suitable environments for the invasive population are suitable for the native population), and expansion (proportion of INV realized niche that does not overlap with AUS realized niche; ranges from 0, when all predicted suitable environments for the invasive population are suitable for the native population, to 1, when no predicted suitable environment for the invasive population is suitable for the native population; expansion = 1-stability) [2]. In addition, the between-class inertia ratio, Schoener’s *D*, Hellinger’s *I*, unfilling, stability, and expansion indexes were calculated to show the difference of the realized niche between New Zealand and Japan populations. We also plotted the frequencies of the climatic conditions for all the climate variables within the distributional ranges (predicted suitable areas) and the total study area (available environment) for AUS and INV, respectively, to reveal the impacts of environmental availability to realized niche shift. In addition, to show the influence of human activity on realized niche shift during invasion, we run a one-way ANOVA for HFP data in the distributional ranges between AUS and INV, and mapped the frequencies of HFP within the distributional ranges and the total study area for AUS and INV based on our full model.

## Results

**Fig. 2:**
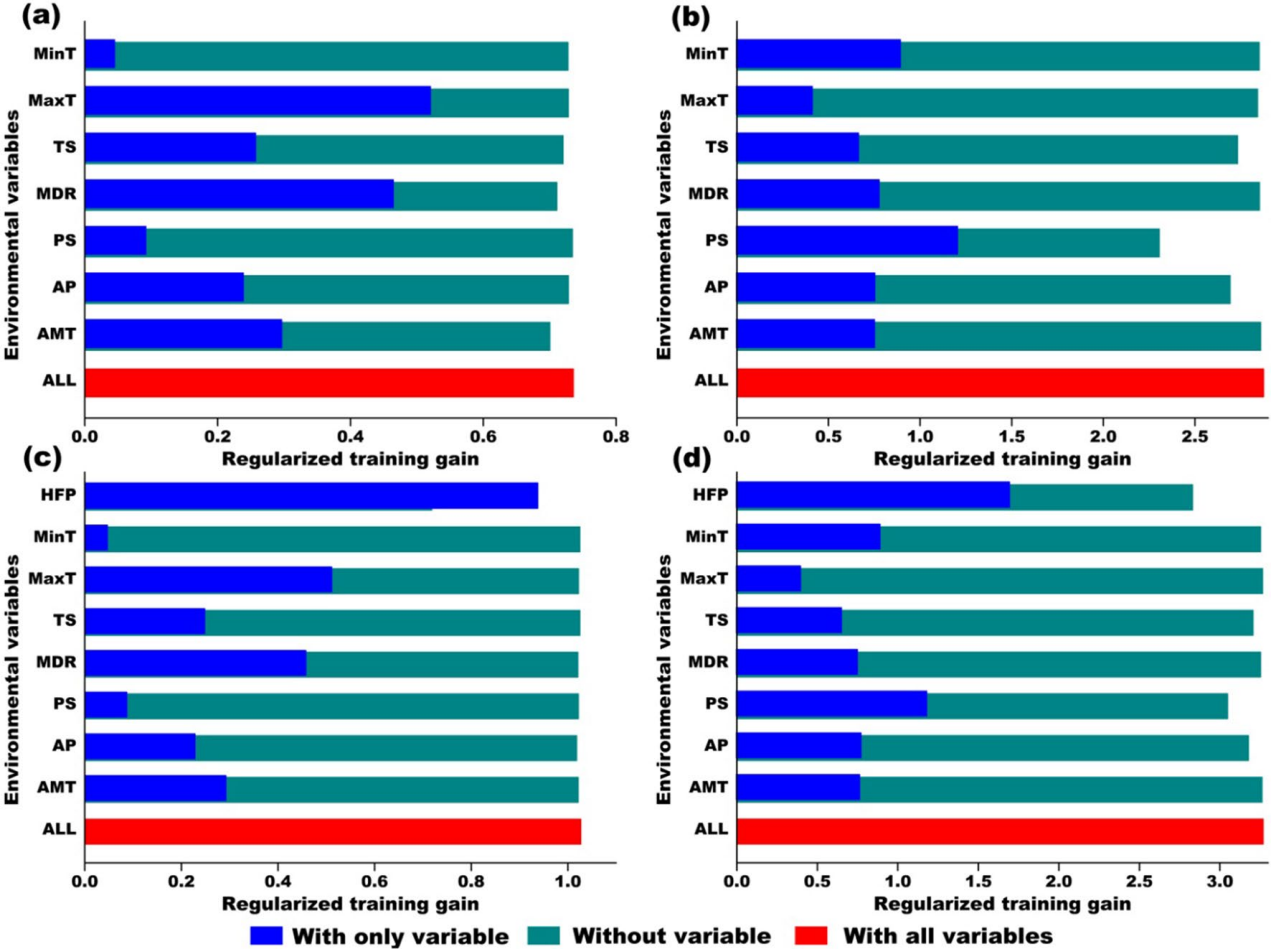
Results of jackknife analyses of the importance of environmental variables for models of *Latrodectus hasselti* distribution. **a** AUS (Australian) climatic model, **b** INV (invasive) climatic model, **c** AUS full model, **d** INV full model. Climatic models include seven variables (see Table 2; Fig. 3). Full models include a measure of human impact index (human footprint) in addition to climatic predictor variables. *AMT* annual mean temperature, *MDR* mean diurnal temperature range, *TS* temperature seasonality, *MaxT* maximum annual temperature, *MinT* minimum annual temperature, *AP* annual precipitation, *PS* precipitation seasonality, *HFP* human footprint

Our ecological niche models (FC = linear, RM = 1, randomkfold partition method: k = 10; climatic model: seven climatic variables, full model: seven climatic variables + HFP) showed good performance for both of the native and invasive populations (Table 1; Table. S2–17 in Additional File 2). Habitat suitability of *L. hasselti* in Australia (native) and the other countries (invasive) based on the climatic and full models are shown in Fig. 1b, c. The models based on all the occurrence records and environmental predictor variables in the study area showed worse performances than the AUS and INV models (lower AUC_test_, higher AUC_diff_, and higher proportions of the occurrence records that had been considered as “absent”; Fig. S1, S2 in Additional File 3). Our model predicted suitable sites for the native *L. hasselti* population in the eastern, western, and southern coasts of Australia. For the invasive populations, in addition to the areas where historical occurrence records were collected (e.g., Japan, New Zealand, eastern part of Papua New Guinea, western coast of India) [66, 101], suitable habitats for the redback spider are mainly located in the southern and eastern regions of China, northwestern regions of South Asia, southern regions of Indo-China Peninsula, eastern regions of Indonesia, and central Philippines (Fig. 1). Our jackknife results revealed that, for the climatic models, the maximum temperature (MaxT) and temperature diurnal range (MDR) have high contributions to the model for the native population, while precipitation seasonality (PS) is most important in the model for the invasive population (Fig. 2). However, human impact had the greatest effects on the models in both areas, if human footprint (HFP) was included in the modeling processes (Fig. 2).

**Table 1:** Performances of the realized niche models of native (AUS) and invasive (INV) populations of *Latrodectus hasselti.* AUC_test_ and AUC_diff_ are showed in mean ± SE

	AUC_test_	AUC_diff_	AICc	AICw
*Climatic model*				
AUS model	0.809 ± 0.022	0.027 ± 0.005	2871.573	0.479
INV model	0.958 ± 0.040	0.022 ± 0.003	706.659	0.563
*Full model*				
AUS model	0.851 ± 0.021	0.021 ± 0.003	2668.910	0.488
INV model	0.973 ± 0.036	0.019 ± 0.002	633.576	0.586

### Realized niche shift

**Fig. 3:**
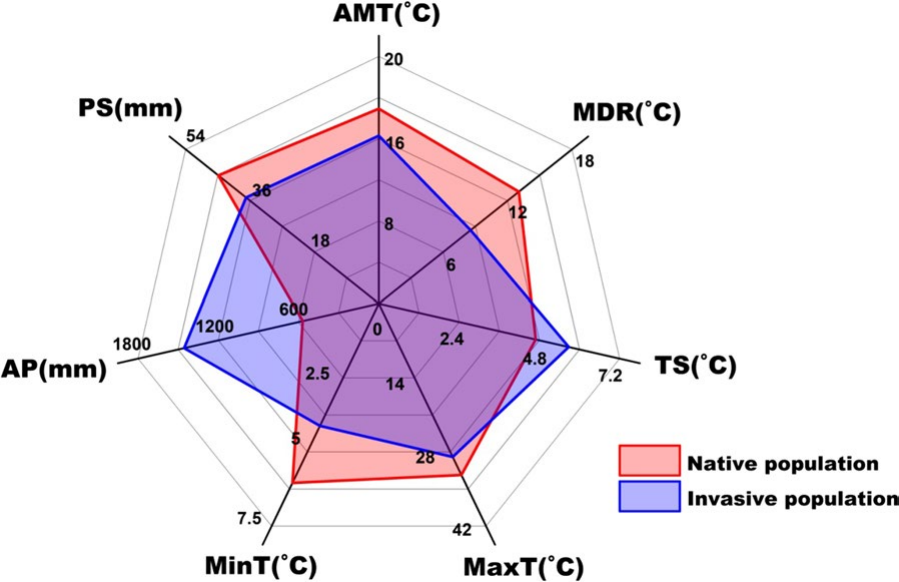
Means of seven climatic variables for native and invasive populations of *Latrodectus hasselti. AMT* annual mean temperature, *MDR* mean diurnal temperature range, *TS* temperature seasonality, *MaxT* maximum annual temperature, *MinT* minimum annual temperature, *AP* annual precipitation, *PS* precipitation seasonality

**Fig. 4:**
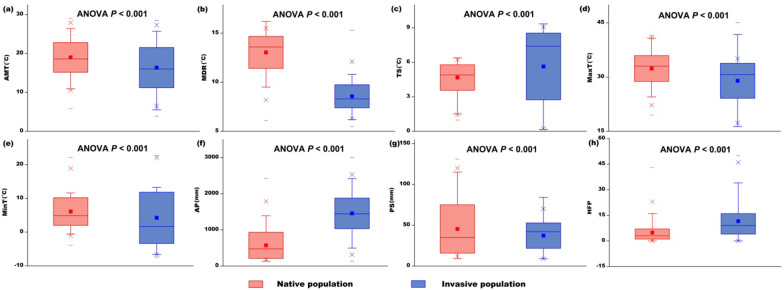
The environmental conditions experienced by native and invasive populations of *Latrodectus hasselti* differ significantly. *AMT* annual mean temperature, *MDR* mean diurnal temperature range, *TS* temperature seasonality, *MaxT* maximum annual temperature, *MinT* minimum annual temperature, *AP* annual precipitation, *PS* precipitation seasonality, *HFP* human footprint

**Fig. 5:**
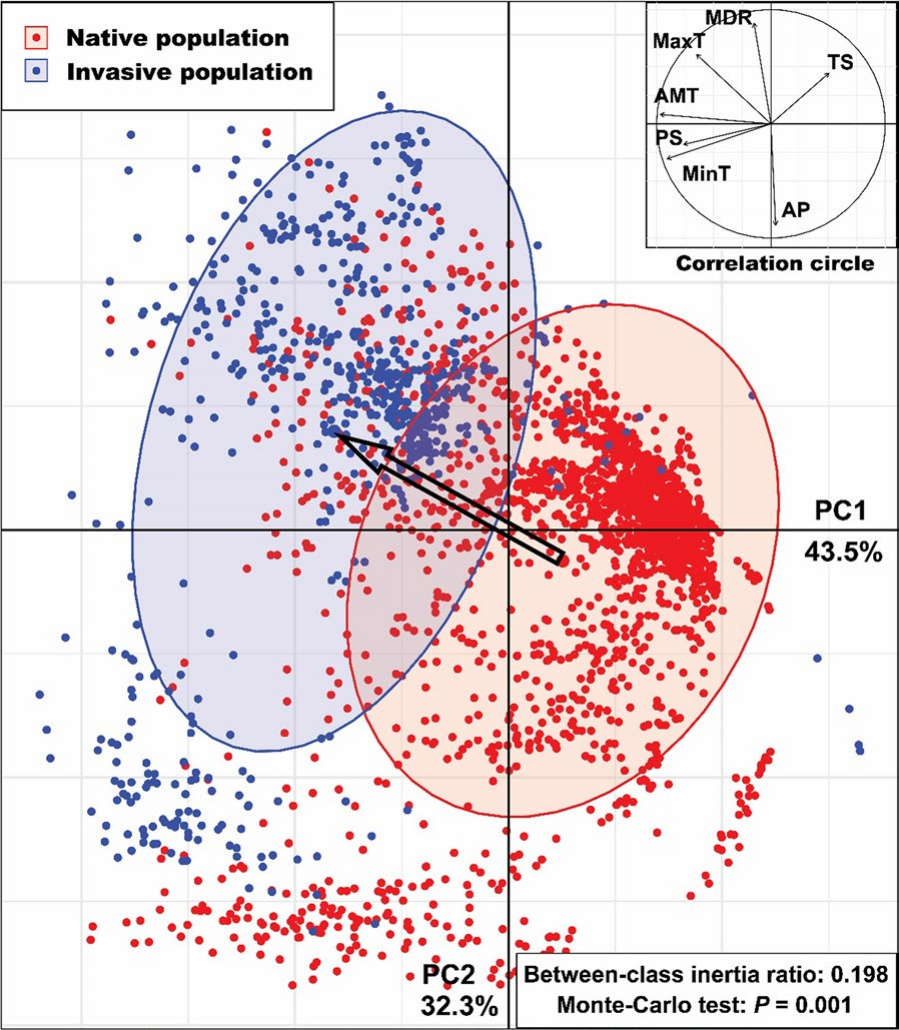
Principal component analysis of climatic predictor variables shows invasive populations of *Latrodectus hasselti* (blue) appear to have had significant climatic realized niche shift in comparison to native populations (red). Open black arrow indicates the direction and magnitude of the shift. *AMT* annual mean temperature, *MDR* mean diurnal temperature range, *TS* temperature seasonality, *MaxT* maximum annual temperature, *MinT* minimum annual temperature, *AP* annual precipitation, *PS* precipitation seasonality

**Fig. 6:**
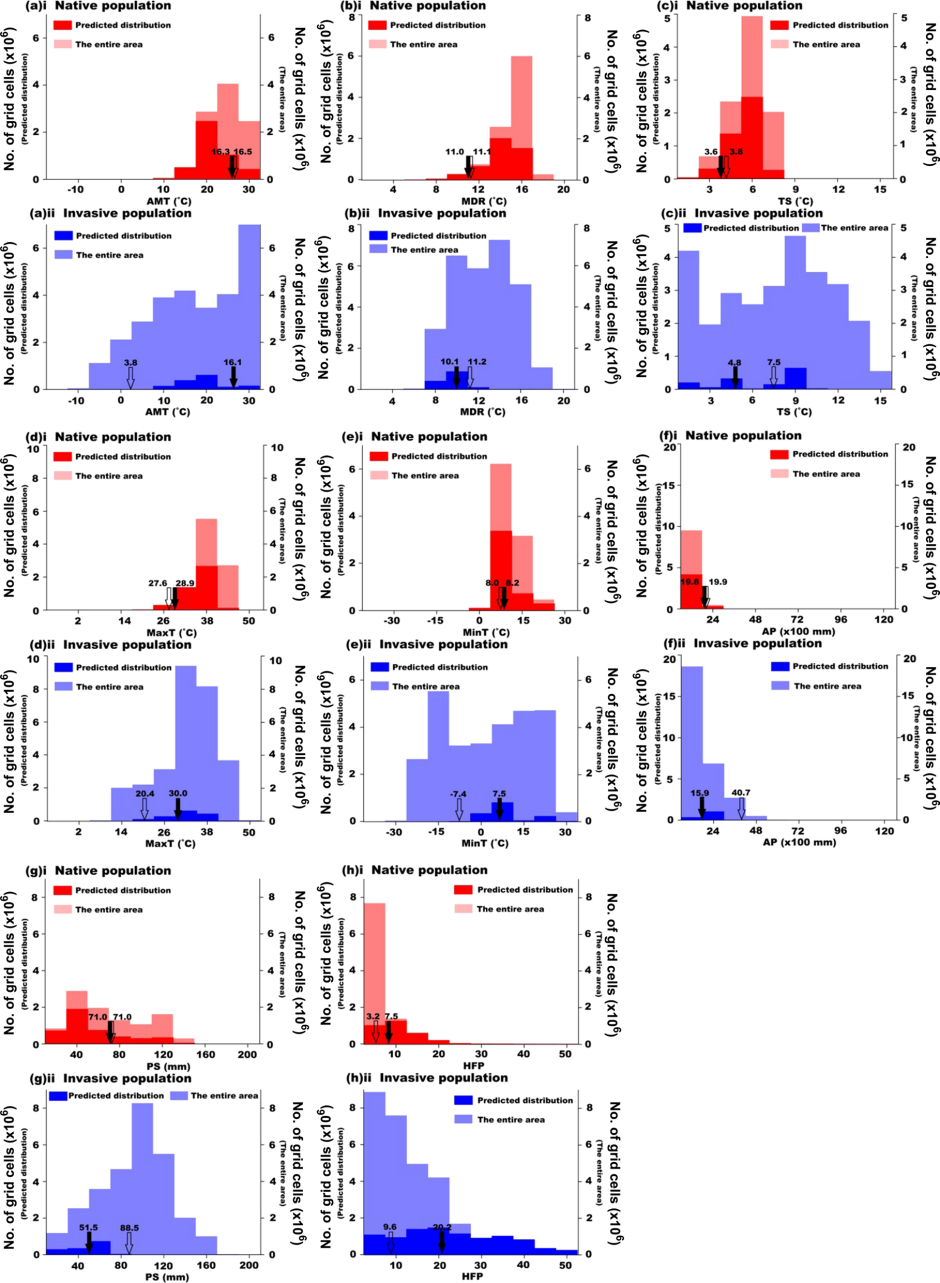
Frequencies of climatic conditions for the eight environmental variables for the predicted distributions (dark colors; for native population, AUS: predicted distribution in Australia; for invasive population, INV: predicted distribution in our study area outside Australia) and the entire study area (light colors; AUS: Australia; INV: our study area outside Australia) of *Latrodectus hasselti* populations (AUS and INV): **a** AMT, **b** MDR, **c** TS, **d** MaxT, **e** MinT, **f** AP, **g** PS, **h** HFP. In each panel, the frequencies for AUS (red colors, i) and INV (blue colors, ii) populations are shown separately. Solid arrows are means for predicted distributions, open arrows are means for the entire study area. *AMT* annual mean temperature, *MDR* mean diurnal temperature range, *TS* temperature seasonality, *MaxT* maximum annual temperature, *MinT* minimum annual temperature, *AP* annual precipitation, *PS* precipitation seasonality, *HFP* human footprint

Significant differences were detected between the means of each climatic variable for the native (AUS) and invasive (INV) habitats (all ANOVA *p* < 0.001; Figs. 3, 4). Our data show that, in their introduced range, redbacks have encountered generally cooler and wetter environments and areas with more seasonal climates than those in the native range (Figs. 3, 4). Two PCs were extracted in our principle component analysis, with a cumulative contribution of 75.8% (PC1: 43.5%, PC2: 32.3%; Fig. 5). The between-class analysis, showed that the realized niche of the species in its invasive range differed from that in its native range, indicating a potential realized niche shift (AUS vs. INV: between-class inertia ratio = 0.198, *p* = 0.001; Fig. 5). The Schoener’s *D* and Hellinger’s *I* indexes showed that similarity or overlap of climatic realized niche between the native and invasive populations were 0.611 and 0.754. For the three niche shift elements, most of the climatic variables showed relatively high stability (values at or approaching the maximum score of 1.00). There were two climatic variables that warrant attention from this analysis: precipitation seasonality (PS, unfilling = 0.138) and temperature seasonality (TS, expansion = 0.672) (Table 2). Therefore, the comparison between the realized niche of the species in its native range and that in its invasive range indicated that there was potential realized niche contraction for precipitation seasonality, and potential realized niche expansion for temperature seasonality. The results revealed that the spiders in New Zealand and Japan may have quite similar realized niches (between-class inertia ratio = 0.937, Schoener’s *D* = 0.947, Hellinger’s *I* = 0.958, for each climatic predictor variable: unfilling < 0.05, stability > 0.95, expansion < 0.05), which indicated that the same niche shift may occur in these two populations compared to the native niche. Frequencies of each climatic variable within the suitable habitats and available habitats in Australia and the other countries were plotted in Fig. 6. Wider ranges of climatic conditions are available in countries in the invasive range of the spider in comparison to those available in Australia (Fig. 6a–g). For the impacts of human activity, we found a significant difference of HFP between AUS and INV populations (ANOVA *p* < 0.001, Fig. 4h). Humans generally have a lower impact in Australia in comparison to the other countries, and the INV population has established in habitats with a greater HFP than where the organism occurs in Australia (Fig. 6h).

**Table 2:** Three elements of niche shift for each climatic variable of *Latrodectus hasselti*

	Niche unfilling	Niche stability	Niche expansion
Annual mean temperature (AMT)	0.003	0.990	0.010
Mean diurnal temperature range (MDR)	0.023	0.995	0.005
Temperature seasonality (TS)	0.000	0.328	0.672
Maximum annual temperature (MaxT)	0.000	0.956	0.044
Minimum annual temperature (MinT)	0.000	0.935	0.065
Annual precipitation (AP)	0.000	0.980	0.020
Precipitation seasonality (PS)	0.138	1.000	0.000

## Discussion

We used an integrative approach that included biologically relevant climatic variables and a human impact metric to compare the realized niches of the native and invasive ranges of the anthropophilic Australian redback spider (*L. hasselti*). Our model predicted suitable sites for both native and invasive *L. hasselti* populations (see Results; Fig. 1). These areas should be emphasized during future field surveys for the species, as they have high environmental suitability. As no or limited field investigations (limited occurrence records) have been carried out, the species could already be present, but may not have been recorded in these areas.

In Australia, temperature variables appear to play an important role in determining the distribution of *L. hasselti* according to our jackknife results (Fig. 2a). That is, native redback spiders are most sensitive to the highest temperature (i.e., MaxT) and temperature variation (i.e., MDR). This may be because warm summers and stable summer temperatures in Australia (e.g., 15–25 °C for 2–3 months) increase successful breeding and offspring hatching, development, and growth of the spiders [62, 66, 101]. However, in the invasive range of *L. hasselti*, precipitation is relatively more important (Fig. 2b), especially precipitation seasonality (i.e., PS), which is followed by the lowest temperature (i.e., MinT). Redback spiders are adapted to low relative humidity (i.e., arid habitats) in their native range, and their development and survival can be impacted by overwintering conditions (including temperature) [61, 66, 101]. Thus, despite the fact that human transport may regularly introduce *L. hasselti* spiders to new areas [60, 66, 73], our model suggests that the likelihood of establishment and spread will heavily depend on local humidity and temperature.

When a human impact metric (i.e., HFP) was included in the modeling, it showed the highest contribution for both native and invasive populations (Fig. 2c, d). Although human influence can lead to over one third of decreases in, and obvious fragmentation of, suitable habitat (see Results, Fig. 1b, c), anthropic environments (e.g., urban areas, human modified habitats) may be beneficial to redbacks by providing shelter from unsuitable climates and extreme climatic events. Thus, human impact may allow redbacks to escape their natural climatic limitations, invade semi-natural ecosystems, and expand their distribution range [61, 66, 69, 73, 101]. The importance of human impact (high HFP, Fig. 2c, d) is consistent with the suggestion of Vink et al. [66] that human-modified environments may support persistence by providing shelter from precipitation and high humidity, and that this is essential for success of arid-adapted redbacks. For example, selecting microhabitats around buildings and structures to build their webs may provide spiders with protection from high and variable precipitation [71]. Moreover, since urban areas are known to be heat islands across seasons [102], high HFP could also buffer invasive redback populations against low winter temperatures. A group of studies on the desert-adapted North American species *Latrodectus hesperus* indicate that populations thrive in urban heat island habitats (within its native range [103–105]). In the case of *L. hesperus*, however, urban spiders experience higher temperatures relative to natural habitats (rather than decreased minimum temperatures, as for redbacks in the invasive range), and this change is associated with reduced survival, poor nutritional condition, but also accelerated development and foraging activity, along with other behavioral changes which may allow persistence [105]. Similarly, recent studies comparing the behavior of invasive and native populations of Australian redback spiders suggest that increased behavioural plasticity in web building, along with increased dispersal and sibling cannibalism, in the invasive range could increase persistence in variable, disturbed habitats [106, 107].

Our study shows that the realized niche of Australian redback spiders may have shifted during or subsequent to their invasion (between-class inertia ratio of first two PCs = 0.198, *p* = 0.001, Fig. 5; Schoener’s *D* = 0.611; Hellinger’s *I* = 0.754). It appears that *L. hasselti* spiders persist in cooler and wetter environments in their invasive range in comparison to those in which they are found within Australia (native population) (Figs. 3, 4, 6). There is further evidence for niche shift in the literature, and it has been reported that redbacks and their spiderlings withstood and survived sub-zero winter temperatures in the field in New Zealand and Japan, which were previously assumed to be unsuitable for them because of the prevalence of low temperatures thought to be fatal [66, 67, 108]. Their occurrences were also recorded in some temperate countries where precipitation was formerly assumed to be too high for redbacks to persist [66]. However, studies to date do not provide concrete evidence suggesting changes in the fundamental niche of redback spiders. Detailed research, especially experimental studies and more empirical field studies, are required in the future. Our results do suggest that the shift in realized niche might be generally or, at least partly, related to the availability of appropriate environmental conditions, as the invasive range has overall lower temperatures, higher precipitation, and greater climatic fluctuations than the native range (Figs. 4, 6). This realized niche shift may also be due to certain environments not being present in part of the spiders’ range. That is, realized niche expansion for temperature seasonality might be due to the fact that the type climates with relatively high temperature seasonality into which expansion has occurred in the other countries do not exist in Australia (Fig. 6c). Consequently, the redbacks in their invasive range occur in environments that are not available in their native range. In addition, our analysis of three elements of niche shift showed that the realized niche shift of *L. hasselti* was mainly related to its expansion along the axes of temperature variation (i.e., temperature seasonality, TS), the contraction along axes of precipitation variation (i.e., precipitation seasonality, PS), but there was generally realized niche conservatism along other climatic variable dimensions (Table 2).

Overall, our results show that human activities have a strong influence on the distribution of redback spiders in their invasive range. That is, in their invasive range the spiders inhabit environments with higher intensities of human activity in comparison to their native range (Fig. 4h). Our data suggest there may be a greater anthropogenic influence overall in the invasive range in comparison to Australia (e.g., high human population in East, South, and Southeast Asia; Fig. 6h). Previous studies in Japan and New Zealand reported that redbacks selected and preferred urban/sub-urban areas (e.g., ditch gratings, rainwater drains, fences, parking lots, cemeteries, buildings), semi-natural habitats (e.g., human-modified coastal sandy beaches and sand dune ecosystems), and artificial structures (e.g., seawalls, revetments, shore line protection structures) [66, 69, 73, 101]. These anthropogenic habitats could provide *L. hasselti* with web-building sites and shelter from extreme (low) temperatures and water stresses (precipitation and humidity), which may outweigh costs from direct or indirect disturbance due to human activities [66, 101]. We infer that the preference of *L. hasselti* for human-disturbed environments, and success in such habitats, may aid survival in new locations with less suitable climatic conditions, and thus underlie potential realized niche shift that enables successful invasion and establishment of this invasive species.

There are a number of factors that should be considered in future research, as they might have influences on the robustness and reliability of the models that predict the potential distributions of invasive species. First, to produce accurate predictions of distribution and realized niche, SDMs need occurrence record datasets with perfect sampling efforts. However, the occurrence records of *L. hasselti* used in this study are mostly from the GBIF and, therefore, most are opportunistically collected. Opportunistically collected occurrence records often contain errors, are biased, and contain gaps. As most of the available occurrence records of redback spiders are from only a few of the countries where the species has been reported (e.g., Australia, New Zealand, Japan, etc.), more field surveys are urgently required for the improvement of the distribution models. Second, this study was carried out at a 1 km × 1 km scale, however, human modified environments could have modulated the climate at finer spatial scales. That means redbacks might not actually experience the climatic conditions measured at larger spatial scales, but their microclimates could not be considered in our model. Third, alien species are often not in equilibrium with their environments in their invasive range and this may lead to niche unfilling. Because they are still spreading, the species may not yet occur in all their suitable environments in the invasive range [109]. Fourth, the impacts of ecological traits of the species (such as behavioral plasticity, dispersal ability) and biological interactions (such as parasites, predators, competitors) should be tested when predicting range expansion of *L. hasselti*, especially for its invasive range. These factors might reduce the possibility and success of invasion, reduce the rate at which the species spreads [109], and exclude the redbacks from climatically suitable habitats.

## Supplementary Information

**Additional file 1. Table S1**: Occurrence records of *Latrodectus hasselti* included in this study.

**Additional file 2. Table S2**: Performances of the AUS climatic models of *Latrodectus hasselti* with the randomkfold (k = 10) partition method. **Table S3**: Performances of the AUS climatic models of *Latrodectus hasselti* with the block partition method. **Table S4**: Performances of the AUS climatic models of *Latrodectus hasselti* with the checkerboard1 partition method. **Table S5**: Performances of the AUS climatic models of *Latrodectus hasselti* with the checkerboard2 partition method. **Table S6**: Performances of the AUS full models of *Latrodectus hasselti* with the randomkfold (k = 10) partition method. **Table S7**: Performances of the AUS full models of *Latrodectus hasselti* with the block partition method. **Table S8**: Performances of the AUS full models of *Latrodectus hasselti* with the checkerboard1 partition method. **Table S9**: Performances of the AUS full models of *Latrodectus hasselti* with the checkerboard2 partition method. **Table S10**: Performances of the INV climatic models of *Latrodectus hasselti* with the randomkfold (k = 10) partition method. **Table S11**: Performances of the INV climatic models of *Latrodectus hasselti* with the block partition method. **Table S12**: Performances of the INV climatic models of *Latrodectus hasselti* with the checkerboard1 partition method. **Table S13**: Performances of the INV climatic models of *Latrodectus hasselti* with the checkerboard2 partition method. **Table S14**: Performances of the INV full models of *Latrodectus hasselti* with the randomkfold (k = 10) partition method. **Table S15**: Performances of the INV full models of *Latrodectus hasselti* with the block partition method. **Table S16**: Performances of the INV full models of *Latrodectus hasselti* with the checkerboard1 partition method. **Table S17**: Performances of the INV full models of *Latrodectus hasselti* with the checkerboard2 partition method.

**Additional file 3. Fig. S1**: Habitat suitability pattern modeled by occurrence records and climatic predictor variables in AUS and INV datasets. Performance of the model were: AUC_test_ = 0.678 ± 0.118, AUC_diff_ = 0.142 ± 0.011 (FC = linear, RM = 1, partition method = randomkfold (k = 10)). The threshold for map conversion (from continuous to binary map) was 0.771. The proportion of the occurrence records that had been considered as “absent” was 20.1%. **Fig. S2**: Habitat suitability pattern modeled by occurrence records, climatic predictor variables, and HFP in AUS and INV datasets. Performance of the model were: AUC_test_ = 0.705 ± 0.098, AUC_diff_ = 0.126 ± 0.010 (FC = linear, RM = 1, partition method = randomkfold (k = 10)). The threshold for map conversion (from continuous to binary map) was 0.722. The proportion of the occurrence records that had been considered as “absent” was 15.7%.

## Data Availability

The datasets used and/or analyzed during the current study are available from the corresponding author on reasonable request.
