# Supplementary material for: Realized niche shift of an invasive widow spider: drivers and impacts of human activities
Source: Front Zool. 2022 Oct 28;19:25. doi: 10.1186/s12983-022-00470-z (PMC9617396; doi:10.1186/s12983-022-00470-z)
Supplement: Supplementary file 3 — Additional file 3. Fig. S1: Habitat suitability pattern modeled by occurrence records and climatic predictor variables in AUS and INV datasets. Performance of the model were: AUCtest = 0.678 ± 0.118, AUCdiff = 0.142 ± 0.011 (FC = linear, RM = 1, partition method = randomkfold (k = 10)). The threshold for map conversion (from continuous to binary map) was 0.771. The proportion of the occurrence records that had been considered as “absent” was 20.1%. Fig. S2: Habitat suitability pattern modeled by occurrence records, climatic predictor variables, and HFP in AUS and INV datasets. Performance of the model were: AUCtest = 0.705 ± 0.098, AUCdiff = 0.126 ± 0.010 (FC = linear, RM = 1, partition method = randomkfold (k = 10)). The threshold for map conversion (from continuous to binary map) was 0.722. The proportion of the occurrence records that had been considered as “absent” was 15.7%. [file 12983_2022_470_MOESM3_ESM.docx]

**Supplementary Information**

**Additional File 3**

**
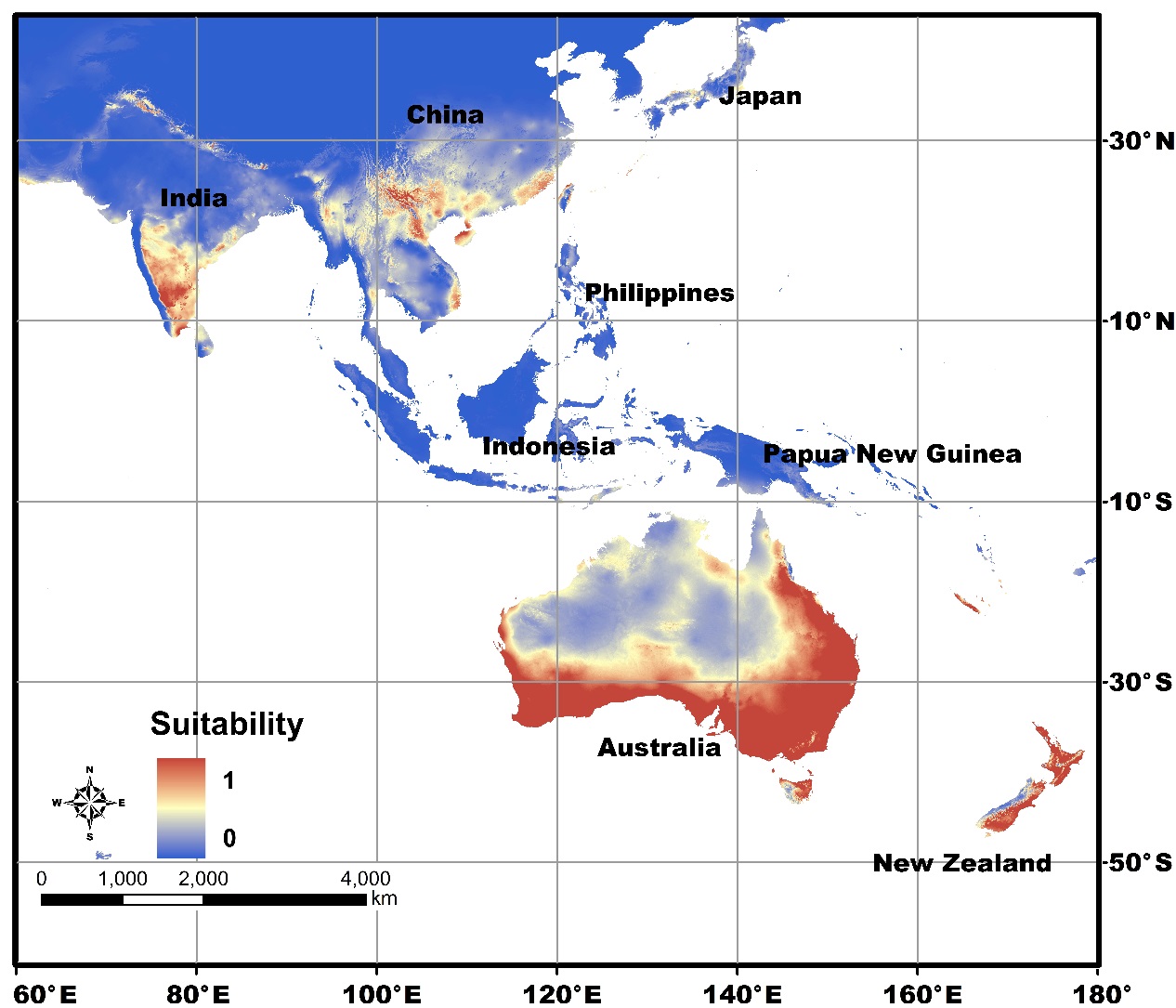
**

Figure S1. Habitat suitability pattern modeled by occurrence records and climatic factors in AUS and INV datasets. Performance of the model were: AUC_test_=0.678±0.118, AUC_diff_=0.142±0.011 (FC=linear, RM=1, partition method=randomkfold (k=10)). The threshold for map conversion (from continuous to binary map) was 0.771. The proportion of the occurrence records that had been considered as “absent” was 20.1%.

**
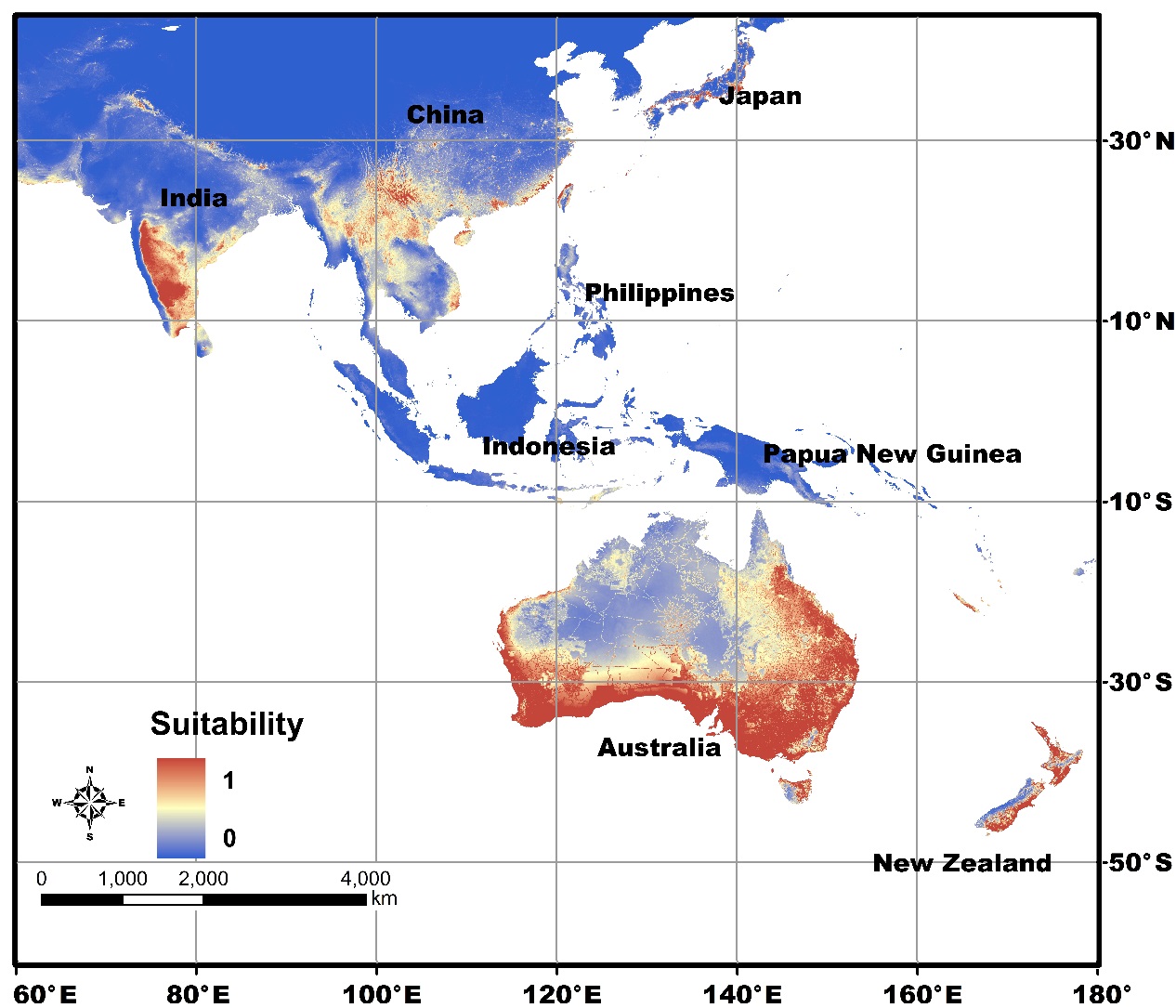
**

Figure S2. Habitat suitability pattern modeled by occurrence records, climatic factors, and HFP in AUS and INV datasets. Performance of the model were: AUC_test_=0.705±0.098, AUC_diff_=0.126±0.010 (FC=linear, RM=1, partition method=randomkfold (k=10)). The threshold for map conversion (from continuous to binary map) was 0.722. The proportion of the occurrence records that had been considered as “absent” was 15.7%.
